# Evaluating the efficacy and safety of Anlotinib in conjunction with stereotactic radiosurgery for small cell lung cancer patients with brain metastases

**DOI:** 10.32604/or.2024.051586

**Published:** 2025-03-19

**Authors:** LIZHI WANG, HUILIN SUN, GUIZHI YU, ZEJING QU, YING JI, YANPING CUI

**Affiliations:** 1 Department of Radiation Oncology, Chengde Central Hospital, Chengde, 067000, China; 2 Department of Hematology, Chengde Central Hospital, Chengde, 067000, China

**Keywords:** Small cell lung cancer (SCLC), Brain metastasis, Anlotinib, Stereotactic radiotherapy, Intracranial progression-free survival

## Abstract

**Background:**

Small cell lung cancer (SCLC) is characterized by its aggressive nature and high propensity for brain metastases. This study investigates the clinical efficacy and safety profile of Anlotinib in combination with Stereotactic Radiotherapy (SRT) for treating brain metastases in patients with small cell lung cancer (SCLC).

**Methods:**

This research included 98 SCLC brain metastasis patients treated at Chengde Central Hospital from October 2020 to January 2024. The patients were categorized into a combined treatment group (CTG) (n = 45) and a Simple SRT group (SSG)(n = 53). The CTG (58 lesions) received Anlotinib with brain SRS, while the SSG (67 lesions) underwent only brain SRS. We compared the rates of intracranial hypertension relief, intracranial lesion treatment efficacy, radiation-induced brain necrosis, intracranial progression-free survival, and overall survival between the groups. Additionally, Anlotinib usage and adverse reactions in the CTG were documented.

**Results:**

Intracranial hypertension relief was significantly higher in the CTG at 80.0% (36/45) compared to 11.3% (6/53) in the SSG (*p* < 0.001). Radiation-induced brain necrosis occurred in 3.4% (2/58) of the CTG, markedly less than the 20.9% (14/67) in the SSG, indicating a significant difference (χ^2^ = 8.479, *p* = 0.004). Effective intracranial lesion treatment rates were 86.7% (39/45) in the CTG and 62.3% (33/53) in the SSG, with a notable difference (χ^2^ = 7.951, *p* = 0.047). The median intracranial progression-free survival was 7.8 months in the CTG *vs*. 4.8 months in the SSG (*p* < 0.0001). Median overall survival times were 11.3 months for the CTG and 7.8 months for the SSG (*p* = 0.3506). The duration of Anlotinib treatment in the CTG was 6 (6, 18) weeks. Adverse reactions included Grade I hypertension in three patients and Grade I hand-foot skin reactions in two patients, with a drug-related adverse reaction rate of 11.1% (5/45).

**Conclusion:**

Anlotinib combined with SRT significantly alleviates brain edema, reduces the incidence of radiation-induced brain necrosis, enhances intracranial progression-free survival, and demonstrates a low adverse reaction rate.

## Introduction

Lung cancer remains the leading cause of cancer-related mortality globally [1] with small cell lung cancer (SCLC) comprising approximately 15% of all cases [2]. Characterized by its high malignancy, aggressiveness, and propensity for brain metastases, SCLC poses significant clinical challenges. At diagnosis, around 10% of SCLC patients already exhibit brain metastases, and this figure escalates to approximately 50% within two years [3,4]. The outlook for these patients is grim, with median survival times lingering at a mere 4–5 months [5]. While current therapeutic strategies include radiotherapy chemotherapy, and surgery therapies, outcomes in terms of survival duration and quality of life remain highly variable. Stereotactic radiotherapy (SRT) has demonstrated superior local control rates for brain metastases when compared to whole-brain radiotherapy (WBRT), offering the benefits of fewer adverse neurological outcomes and reduced treatment sessions without compromising overall survival [6,7].

Based on the latest research advancements, the primary treatment modalities for SCLC include immunotherapy combined with chemotherapy and local therapies. The ASTRUM-005 study is a randomized, double-blind, international, multicenter Phase III clinical trial comparing the efficacy and safety of serplulimab plus chemotherapy *vs*. placebo plus chemotherapy. Interim analysis results, with a median follow-up of 12.3 months, showed that the median overall survival (OS) was 15.4 months in the serplulimab group compared to 10.9 months in the placebo group, extending survival by 4.5 months and significantly reducing the risk of death by 37%. The 24-month OS rates were 43.1% and 7.9%, respectively, and the median progression-free survival (PFS) was 5.7 *vs*. 4.3 months, reducing the risk of disease progression by 52%, with a favorable safety profile [8]. The extended IMpower133 study (IMbrella A) reported a 5-year OS rate of 12% with atezolizumab plus chemotherapy after a median follow-up of 59.4 months [9]. Similarly, the RATIONALE-312 [10] and EXTENTORCH [11] studies demonstrated that immunotherapy combined with chemotherapy significantly extended OS and PFS in patients with extensive-stage SCLC compared to chemotherapy alone, with good tolerability.

WBRT has been the standard treatment for SCLC brain metastases. Patients receiving WBRT typically exhibit more symptoms and higher intracranial disease burden than others [10,12]. WBRT is associated with significant neurocognitive side effects, and current improvements include hippocampal-sparing techniques and the use of memantine to mitigate these adverse effects while maintaining therapeutic efficacy [13–15]. Alternatively, SRT offers a high-precision substitute for WBRT, delivering high-dose radiation to targeted tumor sites with minimal damage to surrounding normal tissues, thereby reducing neurotoxicity and cognitive deficits [16]. Studies comparing WBRT and SRT in SCLC brain metastases show varied results: Chiang et al. [17] included 149 patients with SCLC and brain metastases, revealing that the WBRT group had slightly higher intracranial control rates but lower OS and more intracranial adverse effects compared to the SRT group. The FIRE-SCLC study [18] also evaluated the effectiveness of stereotactic radiosurgery *vs*. WBRT in treating SCLC with brain metastases, showing shorter central nervous system progression times but higher OS in the SRT group compared to the WBRT group.

In the realm of lung cancer treatment, the integration of antiangiogenic agents and SRT is commonplace [19]. Anlotinib is a novel multi-targeted tyrosine kinase inhibitor with anti-angiogenic and anti-tumor properties that help control tumor growth and metastasis. It has shown promising efficacy in various cancers. In small cell lung cancer (SCLC), anlotinib has been evaluated for its potential to improve outcomes when combined with standard therapies. Current studies indicate that anlotinib combined with immunotherapy and/or chemotherapy as a first-or second-line treatment demonstrates favorable results in extensive-stage SCLC, further improving patients’ overall survival (OS) [20–23]. Anlotinib, a multi-targeted tyrosine kinase inhibitor, has proven effective and safe as a third-line or subsequent treatment in SCLC, curtailing tumor angiogenesis and proliferation. It holds the potential to normalize tumor vasculature, enhance local tumor oxygenation, and boost radiosensitivity [24,25]. While the combination of Anlotinib and WBRT has been explored, its efficacy and safety in conjunction with SRT for SCLC brain metastases warrant further investigation. Our study seeks to elucidate and compare the effectiveness and safety of Anlotinib in combination with SRT *vs*. SRT alone in the management of SCLC brain metastases.

## Materials and Methods

### Clinical data

The current study was conducted in accordance with the Declaration of Helsinki and approved by the local ethics committee of the Chengde Central Hospital (CDCHLL2023-407, registered with the Chengde Central Hospital Clinical Trials register). Every patient gave written and informed consent for participation. It involved the analysis of clinical data from 98 patients with SCLC and brain metastases who underwent SRT from October 2020 to January 2024. The cohort was divided into two groups: a combined treatment group (45 patients, 58 lesions) receiving SRT with Anlotinib and an SRT-alone group (53 patients, 67 lesions). Inclusion criteria were SCLC patients with ≤3 brain metastases undergoing SRT, with or without concurrent Anlotinib. Exclusion criteria encompassed previous antiangiogenic therapy before SRT, other antiangiogenic treatments during SRT, prior brain radiotherapy, follow-up periods <10 months, and fewer than two Anlotinib cycles in the combined treatment group.

### Treatment approaches

(1) In the combined treatment group, each patient received SRT for brain metastases. Pre-radiotherapy imaging included Magnetic Resonance Imaging (MRI) and Computed Tomography (CT) scans for localization, using a slice thickness of 1.5 mm. Anlotinib was administered at 12 mg daily for two weeks, followed by a one-week hiatus, constituting a 21-day cycle, throughout the SRT duration. Patients requiring treatment adjustments due to clinical conditions were ensured a minimum of two Anlotinib cycles. (2) Patients in the SRT-alone group underwent SRT for brain metastases, with pre-treatment imaging and positioning mirroring that of the combined group. The SRT protocol adhered to the adaptations based on RTOG 90-05 and 95-08 guidelines [26,27]. According to the Chinese Society of Clinical Oncology (CSCO) guidelines for small-cell lung cancer, for patients with extensive stage small-cell lung cancer: etoposide in combination with cisplatin or carboplatin is the standard regimen for first-line treatment. In addition, irinotecan in combination with platinum-based regimens is also an option for first-line treatment. Immune checkpoint inhibitors targeting PD-1 and PD-L1 have shown promising clinical activity in the treatment of SCLC. In February 2020, China’s National Medicines and Drug Administration (NMPA) formally approved the indication for the first-line treatment of extensive-stage SCLC with the PD-L1 inhibitor atilizumab + etoposide/carboplatin based on the results of the IMpower133 study [28], and subsequent immunological drugs included in the guideline include srulizumab, adalberizumab, and doxorubicin [14–16]. The MDT team was set up in our study centre, and the treatment plan for each patient had to be discussed and decided by the MDT, so the treatment plans of the patients included in this study were basically the same, and all of them underwent standard systemic systemic therapy.

### Observational indicators

The observational indicators encompassed intracranial pressure [29], treatment efficacy, incidence of cerebral radiation necrosis (CRN) [25], intracranial progression-free survival (iPFS), and OS rates. Our diagnostic process for radiation brain necrosis: (1) the patient has a history of brain radiotherapy; (2) when a tumour is found to be enlarged on MRI or neurological symptoms suggestive of tumour progression are found, if we are not sure whether it is a tumour recurrence or radiation brain necrosis, we can perform other advanced brain tumour imaging investigations, such as diffusion-weighted imaging (DWI), perfusion-weighted imaging (PWI), MR spectroscopy (MRS), positron emission tomography (PET) and single photon emission computed tomography (SPECT). Biopsy or surgical excision may be performed if necessary. (3) All results require MDT discussion (consisting of experts in neurology, neurosurgery, neuroradiology, nuclear medicine and radiation oncology) for a final decision on the differential diagnosis of radionecrosis and/or progression. iPFS is defined as the duration from the initiation of radiotherapy to the progression of intracranial tumors or death, whereas OS denotes the time from the beginning of radiotherapy to death from any cause. Additionally, Anlotinib administration and adverse reactions in the combined treatment group were documented.

### Follow-up

The follow-up protocol included monitoring symptoms of intracranial hypertension (evaluated according to CTCAE 5.0 standards) [30,31], conducting routine brain MRIs at 1 month post-treatment, and subsequently every 2–3 months, not exceeding a six-month interval. Immediate reassessment was mandated for patients exhibiting new or aggravated symptoms of intracranial pressure. The assessment of treatment response in brain metastases employed the RANO criteria [32], while CRN detection was based on imaging findings.

### Statistical analyses

Statistical evaluations were conducted using SPSS 27.0. The analyses incorporated Kaplan-Meier survival analysis, log-rank tests, and Cox regression to examine univariate and multivariate factors influencing iPFS. All tests were bidirectional, with a *p*-value of <0.05 denoting statistically significance.

## Results

### General clinical data and pathological characteristics

A total of 98 patients were included in this study, with 45 in the CTG group and 53 in the SSG group. Table 1 presents the general clinical data and pathological characteristics of the patient groups, comparing CTG and SSG. Key characteristics include age, gender, KPS, smoking history, number of BM, BM maximum diameter, extracranial metastasis, and target volume. Notably, the majority of patients in both groups had a single brain metastasis, with 77.8% in the CTG and 77.3% in the SSG. The distribution of patients with two or three brain metastases was also similar between the groups (*p* = 0.758). The size of brain metastases was another critical factor. In the CTG, 53.3% of patients had brain metastases with a maximum diameter of 20 mm or less, whereas in the SSG, 41.5% had metastases of similar size (*p* = 0.242). The presence of extracranial metastases was noted in 73.3% of patients in the CTG and 69.8% in the SSG, indicating a comparable distribution between the groups (*p* = 0.701). There were no statistically significant differences between the groups for the remaining characteristics.

**Table 1:** Comparison of general clinical data of patients in combined treatment group and simple SRT group

Characteristics	CTG N (%)	SSG N (%)	χ^2^	*p*
Years				
<60	19 (42.2)	23 (43.4)		
≥60	26 (57.8)	30 (56.6)	0.014	0.907
Genders				
Male	26 (57.8)	24 (45.3)		
Female	19 (42.2)	29 (54.7)	1.520	0.218
KPS				
<80	18 (40.0)	22 (41.5)		
≥80	27 (60.0)	31 (58.5)	0.023	0.880
Smoking				
Yes	28 (62.2)	27 (50.9)		
No	17 (37.8)	26 (49.1)	1.257	0.262
BM (n)				
1	35 (77.8)	41 (77.3)		
2	7 (15.6)	10 (18.9)		
3	3 (6.6)	2 (3.8)	0.554	0.758
BM maximum diameter				
≤20 mm	24 (53.3)	22 (41.5)		
>20 mm	21 (46.7)	31 (58.5)	1.366	0.242
Extracranial metastasis				
Yes	33 (73.3)	37 (69.8)		
No	12 (26.7)	16 (30.2)	0.148	0.701
Target volume				
≤3 cm^3^	17 (37.8)	30 (56.6)		
>3 cm^3^	28 (62.2)	23 (43.4)	0.188	0.099

### Intracranial hypertension

Before treatment: the median intracranial pressure in the combined treatment group was 18 mmHg (range 15–26 mmHg), and in the monotherapy group, it was 17 mmHg (range 15–24 mmHg), with no statistically significant difference (*p* > 0.05). One month after treatment: the median intracranial pressure in the combined treatment group was 10 mmHg (range 6–19 mmHg), whereas in the monotherapy group, it was 19 mmHg (range 14–31 mmHg), showing a statistically significant difference (*p* < 0.05), see Table S1. In the combined treatment group, 80.0% (36/45) of patients showed improvement in intracranial hypertension symptoms. Specifically, symptoms decreased by two grades in 10 patients (22.2%), by one grade in 26 patients (57.8%), remained unchanged in 5 patients (11.1%), and worsened by one grade in 4 patients (8.9%). Conversely, in the Simple SRT group, symptom improvement occurred in 11.3% (6/53) of patients, with a two-grade decrease in 1 patient (1.9%) and a one-grade decrease in 5 patients (9.4%). Symptoms remained unchanged in 49.1% (26 patients) and worsened in 39.6% (21 patients). The reduction in intracranial hypertension incidence during SRT was significant in the combined treatment group (χ^2^ = 47.036, *p* < 0.001), see Table S2.

### Central radiation necrosis (CRN)

CRN occurred in 3.4% (2/58) of lesions in the combined treatment group, contrasting with 20.9% (14/67) in the Simple SRT group, a difference that was statistically significant (χ^2^ = 8.479, *p* = 0.004), see Table S3. This significant reduction in CRN in the CTG highlights the potential protective effect of Anlotinib when used in combination with SRT. The lower incidence of CRN in the CTG suggests that Anlotinib may mitigate the vascular damage and subsequent necrosis typically induced by radiation therapy. Additionally, the patients in the CTG who did develop CRN experienced less severe symptoms and required less intensive management compared to those in the SSG, further emphasizing the benefit of Anlotinib in reducing the severity of radiation-induced brain injury.

### Efficacy of intracranial lesion treatment

**Figure 1:**
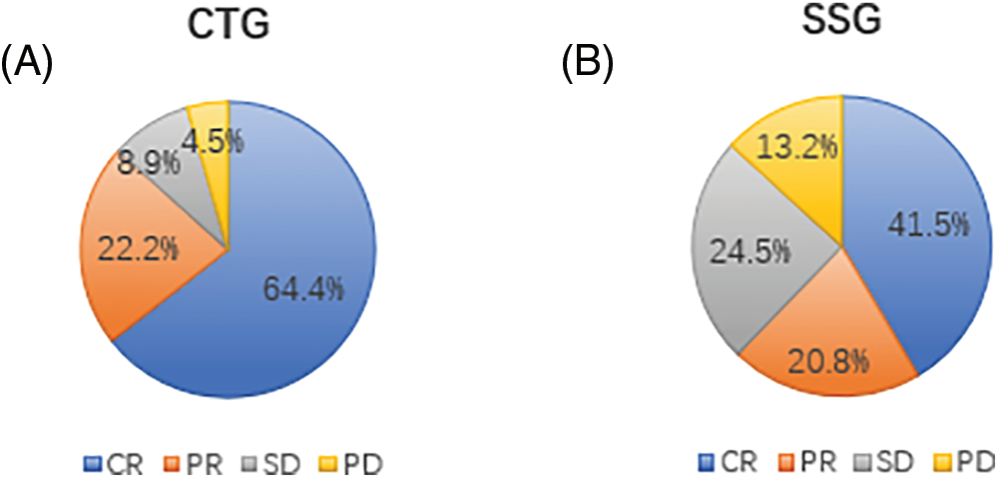
(A) Treatment efficiency of intracranial lesions in the combined treatment group (CTG). The pie chart illustrates the proportion of patients achieving complete remission (CR), partial remission (PR), stable disease (SD), and progression of disease (PD) in the CTG. A total of 64.4% of patients achieved CR, 22.2% achieved PR, 8.9% had SD, and 4.5% experienced PD. This high rate of CR and PR indicates the effectiveness of the combined treatment with Anlotinib and SRT in managing intracranial lesions. (B) Treatment efficiency of intracranial lesions in the simple SRT group (SSG). The pie chart shows the proportion of patients achieving CR, PR, SD, and PD in the SSG. In this group, 41.5% of patients achieved CR, 20.8% achieved PR, 24.5% had SD, and 13.2% experienced PD. Compared to the CTG, the SSG had a lower combined rate of CR and PR, highlighting the superior efficacy of the combined treatment approach.

As per RANO criteria, the combined treatment group saw 64.4% (29 patients) achieve complete remission (CR), 22.2% (10 patients) partial remission (PR), 8.9% (4 patients) stable disease (SD), and 4.5% (2 patients) progression of disease (PD), culminating in an overall treatment efficacy (CR+PR) of 86.6% (39/45) (Fig. 1A). In the Simple SRT group, the rates were 41.5% CR (22 patients), 20.8% PR (11 patients), 24.5% SD (13 patients), and 13.2% PD (7 patients), with an overall efficacy of 62.3% (33/53) (Fig. 1B). The efficacy difference between the groups was statistically significant (χ^2^ = 7.951, *p* = 0.047). We further compared the intracranial treatment efficacy (SD+PD) between patients in the combination therapy group and the SRT alone group, and the combination therapy group was significantly better than the treatment alone group (χ^2^ = 7.435, *p* = 0.006) (Table 2).

**Table 2:** Comparison of intracranial treatment efficacy between the combined treatment group and the SRT alone group

Therapeutic effect	Combined treatment group N (%)	Simple SRT group N (%)	χ^2^	*p*
CR+PR	39 (86.7)	33 (62.3)		
SD+PD	6 (13.3)	20 (37.7)	7.435	0.006

### Intracranial progression-free survival (iPFS)

**Figure 2:**
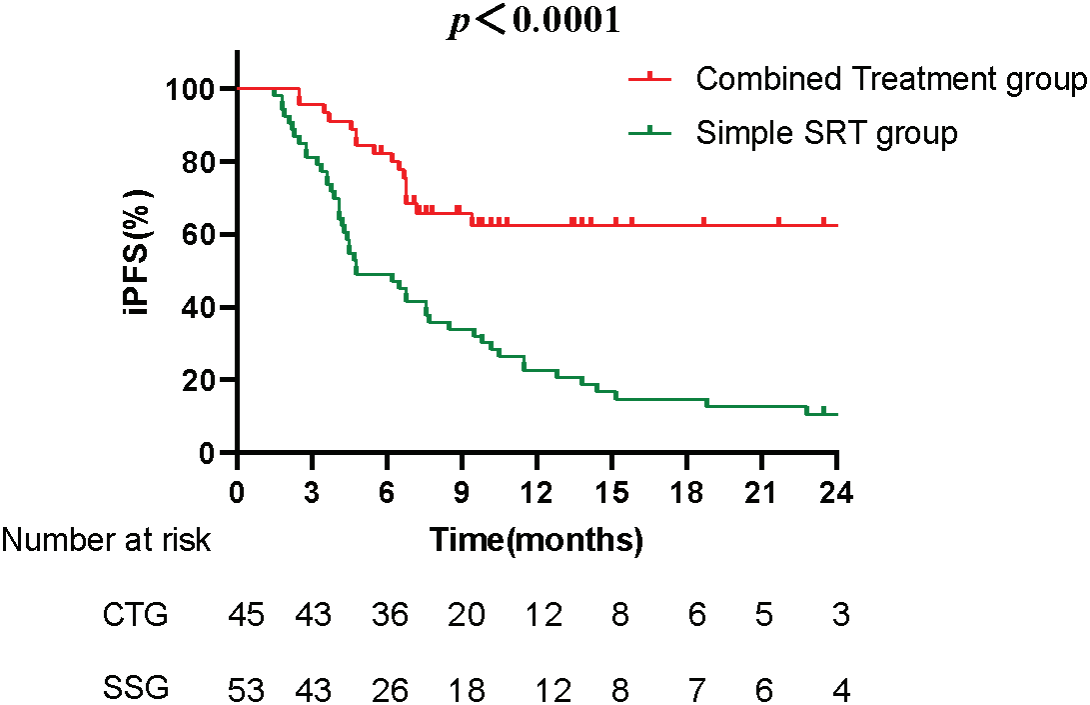
The Kaplan-Meier survival curves compare the iPFS between the combined treatment group (CTG) and the simple SRT group (SSG). The median iPFS for the CTG was 7.8 months, significantly longer than the 4.8 months observed in the SSG (*p* < 0.0001). The curve for the CTG remains higher over time, indicating prolonged periods without disease progression for patients receiving the combined treatment.

The median iPFS was 7.8 months in the combined treatment group compared to 4.8 months in the Simple SRT group, a statistically significant difference (*p* < 0.0001) (Fig. 2). Univariate and multivariate analyses identified having more than one brain metastasis, a maximum brain metastasis diameter greater than 20 mm, and the absence of Anlotinib treatment as independent risk factors influencing iPFS (Table 3).

**Table 3:** Analysis of factors influencing iPFS in 98 patients with brain metastases from small cell lung cancer

Characteristics	Univariate analysis			Cox multifactorial analysis		
	HR	95% CI	*p*	HR	95% CI	*p*
Years						
<60	1.000					
≥60	1.063	0.668–1.691	0.797			
Genders						
Male	1.000					
Female	1.217	0.768–1.929	0.403			
KPS						
≥80	1.000					
<80	1.240	0.777–1.977	0.367			
Smoking						
Yes	1.000					
No	1.231	0.771–1.964	0.384			
BM (n)						
1	1.000		0.001	1.000		<0.001
2	2.583	1.466–4.554	0.001	2.415	1.368–4.265	0.002
3	2.785	1.100–7.055	0.031	3.700	1.388–9.863	0.009
BM maximum diameter						
≤20 mm	1.000			1.000		
>20 mm	1.686	1.053–2.701	0.030	1.665	1.007–2.751	0.047
Extracranial metastasis						
Yes	1.000					
No	1.394	0.877–2.214	0.160			
Target volume						
≤3 cm^3^	1.000					
>3 cm^3^	1.537	0.940–2.513	0.087			
Anlotinib						
Yes	1.000			1.000		
No	2.164	1.332–3.515	0.002	2.023	1.238–3.304	0.005

### Overall survival (OS)

**Figure 3:**
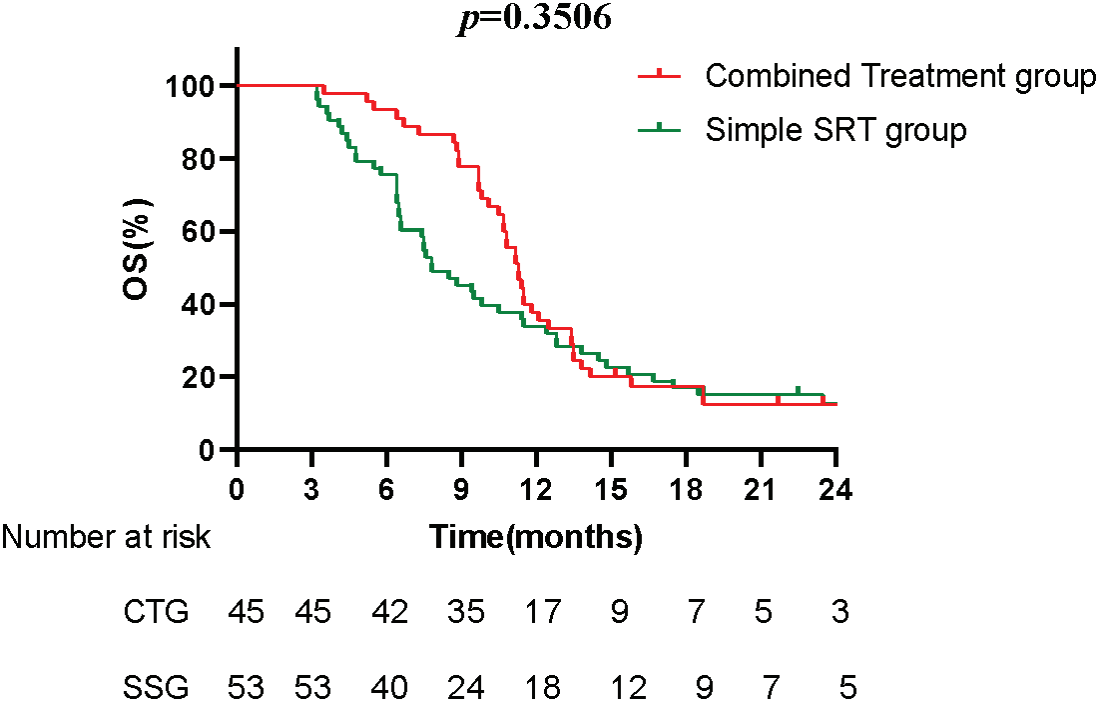
The Kaplan-Meier survival curves compare the overall survival between CTG and the SSG. The median OS for the CTG was 11.3 months, while the SSG had a median OS of 7.8 months. Although the difference in OS was not statistically significant (*p* = 0.3506), the trend suggests a potential survival benefit with the combined treatment.

The Kaplan-Meier survival curves compare the overall survival between the CTG and the SSG. The median OS for the CTG was 11.3 months, while the SSG had a median OS of 7.8 months. Although the difference in OS was not statistically significant (*p* = 0.3506), the trend suggests a potential survival benefit with the combined treatment (Fig. 3).

### Anlotinib usage and adverse reactions in the combined treatment group

Anlotinib was administered for a median of 6 weeks (range 6–18 weeks), with treatment cycles occurring every 3 weeks. In the combined treatment group, Grade I hypertension occurred in three patients (6.7%), and Grade I hand-foot skin reactions were observed in two patients (4.4%), resulting in a drug-related adverse reaction rate of 11.1% (5/45). No severe (Grade II or higher) adverse reactions were reported, indicating a favorable safety profile. The low incidence and mild nature of adverse reactions suggest that Anlotinib is well-tolerated when used with SRT, without requiring treatment discontinuation. This tolerability is crucial for maintaining the overall efficacy of the combined treatment regimen for patients with small cell lung cancer and brain metastases.

## Discussion

The treatment approaches for brain metastases in SCLC are becoming increasingly diverse. Chiang et al. [17] divided patients with SCLC and brain metastases into three groups: those receiving systemic therapy alone, those receiving SRS combined with systemic therapy, and those receiving WBRT combined with systemic therapy. The primary outcomes were OS and time to central nervous system progression (TTCP). The results showed that 48 patients (32.2%) received systemic therapy alone, 33 received SRS combined with systemic therapy, and 68 received WBRT combined with systemic therapy. The median OS and TTCP were 7.2 months and 8.7 months, respectively. The OS and TTCP for the systemic therapy alone group, the WBRT group, and the SRS group were 7.7 months and 9.3 months, 4.2 months and 14.1 months, and 8 months and 4.5 months, respectively. The main prognostic factors affecting OS were age, number of brain metastases, and use of chemotherapy and immunotherapy. This study indicates that while the WBRT group demonstrated better intracranial control, it had worse OS and caused neurocognitive damage. Therefore, for SCLC patients with brain metastases, treatment decisions should consider prognosis, physical condition, and the number of brain metastases.

Although traditionally it was believed that immunotherapy drugs could not effectively cross the blood-brain barrier, recent studies have shown that immunotherapy can benefit patients with brain metastases from lung cancer. This may be due to the activation of the immune system and increased microvascular permeability following immunotherapy, allowing activated T cells to penetrate the blood-brain barrier and exert antitumor effects intracranially [33]. The CASPIAN study [34] compared the prognosis of durvalumab combined with chemotherapy *vs*. chemotherapy alone in SCLC patients with brain metastases. The combined group and the chemotherapy group included 28 (10.4%) and 27 (10.0%) patients, respectively. Results showed that durvalumab combined with chemotherapy tended to improve OS (HR = 0.79, 95% CI: 0.44–1.41) and PFS (HR = 0.73; 95% CI: 0.42–1.29) compared to chemotherapy alone. Notably, the CASPIAN study design only allowed PCI for brain metastasis patients in the control group, not in the immunotherapy group. Despite this, durvalumab showed a trend toward OS benefit in the brain metastasis population. However, this analysis is limited by the small sample size, necessitating larger prospective studies for validation. In contrast, the IMpower133 study [28] showed no significant OS benefit for atezolizumab in SCLC patients with brain metastases (HR = 1.07, 95% CI 0.47–2.43). The ASTRUM-005 study [8] reported an HR of 0.73 (95% CI 0.42–1.25) for brain metastasis patients in the immunotherapy group. Based on these studies, there is no consensus on whether immunotherapy improves prognosis in SCLC patients with brain metastases, but combining immunotherapy with intracranial radiotherapy may offer benefits.

Extensive research has been conducted on Anlotinib combined with radiotherapy for non-small cell lung cancer (NSCLC) brain metastases, showing favorable clinical outcomes [35]. However, literature on its use in SCLC brain metastases, particularly in the oligometastatic setting, is limited, with a notable gap in domestic studies. This study evaluates the effectiveness of Anlotinib with SRT in treating oligometastatic SCLC brain metastases. Findings suggest that Anlotinib can mitigate intracranial hypertension, decrease the incidence of CRN, and prolong the iPFS in SCLC brain metastasis treatment with SRT.

Anlotinib’s antiangiogenic properties, which reduce vascular permeability caused by tumor invasion, likely contribute to the diminished adverse reactions and improved therapeutic outcomes observed in the combined treatment group [36]. Radiation therapy can damage vascular tissues, leading to hypoxia and the upregulation of HIF, which, in turn, prompt reactive astrocytes to release VEGF. Elevated VEGF levels foster abnormal neovascularization, exacerbating brain edema by increasing vascular permeability. Increased local intracranial pressure is often caused by cerebral edema, which in turn causes local ischemia and hypoxia in the brain tissue, which is a vicious circle, and eventually even develops into radiation brain necrosis [37]. Anlotinib, a multi-targeted tyrosine kinase inhibitor, acts on VEGFR2/3, FGFR1-4, PDGFRα/β, c-Kit, and Ret [38], potentially reducing intracranial pressure during SRT. Zhuang et al. [39] studied the efficacy of anlotinib in patients with NSCLC brain metastases with brain edema after receiving anti-PD-1/PD-L1 therapy. The inclusion criteria were stage IV NSCLC, treatment with anti-PD-1/PD-L1 inhibitors, and the presence of brain metastases with brain edema. Anlotinib was started one week before SRT, and the endpoints were the brain edema index and intracranial hypertension symptoms before and after anlotinib use. The results showed that anlotinib significantly reduced the brain edema index of 23 brain edema lesions, from 7.87 ± 3.34 to 3.19 ± 1.45 (*p* < 0.01); besides improving edema, anlotinib also effectively alleviated intracranial hypertension symptoms in patients. Before anlotinib treatment, the median grade of intracranial hypertension symptoms (grades 0 to 2) was 2, significantly improved to grade 1 after anlotinib treatment (*t* = 7.5, *p* < 0.01). During anlotinib treatment, SRT did not cause significant intracranial hypertension symptoms, indicating that anlotinib can effectively reduce brain edema caused by NSCLC brain metastases. The study by Wang et al. [40] showed that the intracranial response rate in the combination therapy group was 71.4% (15/21), higher than the 12.0% (3/25) in the single SRS group (*p* < 0.001). Antiangiogenic therapy might also facilitate vascular normalization, curbing brain edema and ischemia [41], In addition, studies have shown that vascular injury may be a major cause of CNS toxicity from radiotherapy. Antivascular therapy promotes vascular normalization, thereby reducing brain edema and ischemia and hypoxia in the tumor and surrounding tissues [42], and possibly reducing CRN occurrence [43]. On the other hand, antivascular therapy can increase the blood supply and oxygenation of the tumor, which theoretically can increase the sensitivity of radiotherapy and thus improve the efficacy of radiotherapy. And the difference in the treatment efficiency of intracranial lesions between the two groups of patients in this study was statistically significant, which is consistent with previous studies. Wang et al. [40] included 46 patients with NSCLC accompanied by brain metastases. Among them, 21 patients were in the anlotinib combined with SRT group, and 25 patients were in the SRT alone group. The treatment efficacy for intracranial lesions in the two groups was 80.9% (17/21) and 60.0% (15/25), respectively, with no statistically significant difference (*p* = 0.289). In this study, the treatment efficacy in the combined group was 86.7% (39/45), while in the SRT alone group, it was 62.3% (33/53), with a statistically significant difference between the two groups (χ^2^ = 7.951, *p* = 0.047). Comparing the two studies, it was found that whether in the combined treatment group or the SRT alone group, the results of the two studies were very similar. However, Wang et al.'s study showed no statistically significant difference in treatment efficacy between the two groups. The analysis suggested that this might be due to the smaller number of cases included in their study, all of which were patients with NSCLC, including squamous cell carcinoma, adenocarcinoma, and other pathological types, leading to differences in sensitivity to SRT, thus resulting in an overall lower treatment efficacy compared to this study. He et al. [44] studied the effectiveness of anlotinib combined with brain radiotherapy in the treatment of brain metastases from NSCLC. The results showed that the objective response rate (ORR) in the 28 patients treated with combination therapy was 89.29%, higher than the 80.0% in the 45 patients receiving radiotherapy alone. The ORR in the monotherapy group of this study was higher than that reported in their study. The analysis suggested that this could be attributed to the inclusion of patients receiving whole-brain radiotherapy combined with local radiotherapy in the monotherapy group of their study (17.78%), leading to a higher ORR. In another retrospective study [45], the application of other anti-angiogenic agents such as bevacizumab in the treatment of brain metastases from lung cancer was evaluated. The study assessed the effectiveness and safety of SRT combined with bevacizumab in treating brain metastases primarily from lung and breast cancer. The results showed that compared to the radiotherapy alone group, the combination therapy group had better efficacy (84% *vs*. 51%, *p* = 0.001). This is consistent with the results of our study.

The EORTC study suggested that PCI could improve survival and reduce the probability of late brain metastases in patients with extensive stage SCLC after systemic treatment with good efficacy (CR/PR) [46], whereas the Japanese randomised controlled study suggested that PCI could reduce the probability of intracranial metastases when intracranial metastases were ruled out by NMR brain testing (48% *vs*. 69%, *p* < 0.0001), but did not provide a survival benefit [47], therefore, PCI in patients with extensive stage SCLC should be considered in a comprehensive manner. *vs*. 69%, *p* < 0.0001), but it did not provide a survival benefit [6], therefore, PCI in patients with extensive stage SCLC should be decided carefully after considering the patient’s condition. The patients included in this study did not undergo PCI for the following reasons: (1) we considered that PCI might affect the results of this study, such as patient prognosis, intracranial adverse effects, etc., resulting in biased results; (2) in clinical practice, there are a part of patients who could not tolerate or refused to undergo PCI, and this study mainly included these patients; (3) the current consideration that PCI can cause neurocognitive impairment, and whether it can improve the survival of extensive-stage SCLC. The prognosis of patients with extensive stage SCLC is still controversial, especially in the era of MRI, so the application of PCI in patients with extensive stage SCLC is becoming more and more controversial, and the present study mainly included the prognosis and safety of patients who did not undergo PCI after brain metastasis and brain radiotherapy with or without amilorotinib, which also provides a certain basis for the value of the application of PCI in extensive stage SCLC. The study also provides some basis for the value of PCI in extensive stage SCLC.

Despite the promising results, this study has several limitations. First, the sample size was relatively small, which may limit the generalizability of the findings. Second, the study was conducted at a single center, and the results might not be applicable to other settings with different patient populations or treatment protocols. Third, the retrospective nature of the study introduces potential biases in data collection and analysis. Finally, the follow-up period was relatively short, and longer-term outcomes such as late toxicity and overall survival beyond the study period were not fully assessed. Future studies with larger, multi-center cohorts and longer follow-up periods are necessary to validate these findings and further elucidate the long-term benefits and risks of combining Anlotinib with SRT for the treatment of brain metastases in SCLC patients.

## Conclusions

The present study demonstrated that Anlotinib could extend iPFS, possibly by targeting latent intracranial metastases. Analysis identified the absence of Anlotinib as an independent risk factor for shorter iPFS. The safety profile of Anlotinib in combination with SRT in this context appears favorable, with low adverse reaction rates, potentially due to the brief treatment duration (most patients received two cycles). However, the retrospective nature and small sample size of this study call for further confirmation through larger, multicenter prospective studies. In conclusion, Anlotinib with SRT may alleviate intracranial hypertension, enhance treatment tolerability, lower CRN incidence, and increase iPFS in SCLC brain metastasis management.

## Supplementary Materials





## Data Availability

All data generated or analysed during this study are included in this article. Further enquiries can be directed to the corresponding author.

## Abbreviations

SSG: Simple SRT group
CTG: Combined treatment group
SRT: Stereotactic radiotherapy
SCLC: Small cell lung cancer
WBRT: Whole brain radiotherapy
CRN: Central radiation necrosis
iPFS: Intracranial progression free survival
OS: Overall survival
CR: Complete remission
P: Partial remission
SD: Stable disease
PD: Progression of disease

## Supplementary Materials

The supplementary material is available online at https://doi.org/10.32604/or.2024.051586.
