# Supplementary material for: Evaluating the efficacy and safety of Anlotinib in conjunction with stereotactic radiosurgery for small cell lung cancer patients with brain metastases
Source: Oncol Res. 2025 Mar 19;33(4):885–94. doi: 10.32604/or.2024.051586 (PMC11964871; doi:10.32604/or.2024.051586)
Supplement: Supplementary file 1 [file OncolRes-33-51586-s001.docx]

Table S2: Comparison of improvement of intracranial hypertension symptoms in patients in CTG and SSG groups

| Intracranial hypertension symptoms | Combined treatment group  N (%) | Simple SRT group  N (%) | χ² | *p* |
| --- | --- | --- | --- | --- |
| Decreased by two grades | 10 (22.2) | 1(1.9) |  |  |
| Decreased by one-grade  Unchanged  Worsened | 26(57.8)  5(11.1)  4(8.9) | 5 (9.4)  26(49.1)  21(39.6) | 47.036 | ＜0.001 |

Table S3: Comparing the incidence of CRN in patients in the CTG and SSG groups

| Central Radiation Necrosis | CTG  N (%) | SSG  N (%) | χ² | *p* |
| --- | --- | --- | --- | --- |
| Yes | 2 (3.4) | 14 (20.9) |  |  |
| No | 56 (96.6) | 53 (79.1) | 8.479 | 0.004 |
